# Analysis of dietary pattern effects on metabolic risk factors using structural equation modeling

**DOI:** 10.3389/fnut.2025.1540919

**Published:** 2025-06-25

**Authors:** Åse Mari Moe, Sigrunn H. Sørbye

**Affiliations:** Department of Mathematics and Statistics, UiT The Arctic University of Norway, Tromsø, Norway

**Keywords:** cross-sectional study, CVD risk factors, dietary patterns, exploratory structural equation models, mediation analysis, Tromsø Study

## Abstract

**Background:**

This study aimed to investigate the effects of dietary patterns on metabolic cardiovascular disease (CVD) risk factors in a Nordic population.

**Methods:**

The study sample comprised 9,988 participants aged 40–79 years from the seventh Tromsø study (Norway). Available data included food intake values collected by a food frequency questionnaire. Exploratory structural equation models were utilized to analyse direct, indirect, and total effects of dietary patterns on metabolic CVD risk factors, using obesity as a mediator. The CVD risk factors included CRP, HDL-cholesterol, triglycerides, glycated hemoglobin, and blood pressure. All structural equations were adjusted for available lifestyle and demographic variables.

**Results:**

Three common dietary patterns for women and men were identified, named Snacks and Meat, Health-conscious, and a Processed Dinner pattern. Additionally, a Porridge pattern was identified for women and a Cake pattern for men. The Health-conscious pattern showed a direct favorable effect on HDL-cholesterol (both sexes) and triglycerides (women). The Snacks and Meat pattern showed an unfavorable direct effect on triglycerides (men), while the Cake pattern had a favorable effect. All patterns, except the Health-conscious pattern for women, had direct effects on obesity, indirect effects on all metabolic risk factors, and a total effect on CRP. Snacks and Meat and the Processed Dinner patterns had unfavorable total effects on HDL-cholesterol (both sexes).

**Conclusion:**

Dietary patterns showed direct associations with HDL-cholesterol and triglycerides. Obesity was an important mediator in explaining the indirect effects of dietary patterns on all metabolic risk factors.

## 1 Introduction

Lifestyle variables such as diet, smoking, alcohol consumption and physical inactivity, have been identified as important individual risk factors in developing cardiovascular disease (CVD), a leading cause of mortality worldwide (1). Specifically, an unhealthy diet is a recognized and significant contributor to obesity which is well-known to induce disturbances in cardiovascular and metabolic functions in the body (2–4). In addition to obesity, important metabolic CVD risk factors include dyslipidemia, hypertension and insulin resistance (5). Chronic inflammation, as measured by increased levels of C-reactive protein (CRP), has also been suggested as a risk factor, not merely as a risk marker (6).

The interplay between individual lifestyle variables and metabolic CVD risk factors is complex. Obesity is closely related to dietary factors (7), while also being an individual metabolic CVD risk factor (8). In modeling the associations between dietary patterns and components of metabolic syndrome, the influence of diet was most pronounced in estimating waist circumference, a widely used measure of obesity (9). Estimated associations between diet and metabolic risk factors beyond obesity typically change depending on whether the analysis is adjusted for body mass index (BMI) or not (10, 11).

Due to the strong associations between diet and obesity, it is natural to model obesity as a mediator, partially explaining some of the effects of diet on metabolic risk factors (12, 13). However, only a few analyses have used models in which obesity acts as a mediator and investigated the direct, indirect and total effects of diet on recognized metabolic risk factors (14). Estimation of these effects is easily biased unless the models adjust for potential confounders, like lifestyle and demographics variables. For example, socioeconomic status has shown strong links to both diet and metabolic risk factors (15).

The convenient framework of exploratory structural equation models (ESEM) facilitates assessment and testing of assumed relationships between variables, allowing for inclusion of mediators, confounders and incorporation of prior knowledge. ESEM combines exploratory factor analysis (EFA) with structural equation models (SEM) (16). The structural SEM part allows for simultaneous estimation of all associations of a hypothesized model using regression equations (17). This structural part can be used to estimate associations between dietary patterns, obesity and the observed CVD risk factors, adjusting for confounding variables. The EFA part is simultaneously used to construct latent dietary patterns based on observed intake values of given food variables (14). Often, such dietary patterns are constructed prior to the statistical analysis, while ESEM combines this with structural regression analysis. This allows for dietary patterns to overlap, making ESEM more flexible compared to standard SEM (16, 17).

The primary aim of the given study was to investigate the direct, indirect and total effects of dietary patterns on established metabolic CVD risk factors using ESEM. The analysis was conducted on data from the seventh survey of the Tromsø Study including 9,988 participants. The study sample can be seen to represent a general Nordic population, and the large sample size facilitated separate analysis for women and men.

## 2 Materials and methods

### 2.1 Study population

The Tromsø Study is a comprehensive population-based health study conducted in the municipality of Tromsø, Norway. Currently, it includes a total of 7 consecutive cross-sectional surveys (Tromsø1–Tromsø7) conducted within the years 1974 to 2016. In total, more than 45,000 individuals have participated in one or more of these surveys. In addition to questionnaires and interviews, collected data included clinical measurements and biological samples (18).

### 2.2 Study sample

Our study sample included participants of Tromsø7 who also answered a comprehensive food frequency questionnaire (FFQ). The survey was conducted in 2015–2016 and originally all inhabitants of Tromsø municipality above the age of 40 were invited to participate. Out of the 21,070 individuals who participated in Tromsø7 and consented to research, 15,139 answered the FFQ. Of these, 3,487 answered less than 90% of the FFQ and were excluded. Further, 405 did not report on physical activity level, education level, or smoking status. Additionally, 205 had missing measurements on height, weight, waist circumference, blood pressure, hemoglobin A1c (HbA1c), HDL-cholesterol or C-reactive protein (CRP). All of these 610 participants were excluded. Also, 393 participants were excluded due to having either the 1% highest or lowest total energy intake or the 1% highest or lowest total water intake. Finally, participants of 80 years of age or older, and participants with known diabetes were excluded. Participants who did not provide information on diabetes or prior diabetes were treated as without diabetes. The final study sample included 9988 respondents (Supplementary Figure S1).

### 2.3 Dietary data

The paper-based FFQ of Tromsø7 has been validated in (19) and further detailed in (20). After completing the form, the participants returned it by postal mail using a pre-paid envelope and answers were checked manually by trained technicians before scanning. The FFQ included a total of 261 questions, providing individual average intake values of various dishes, foods, beverages and supplements. The responses to the FFQ were used to calculate the food intake in g/day using the food composition database and nutrient calculation system KBS. The questions on food intake were aggregated into 35 food variables, excluding supplements, water, tea and alcohol consumption (Supplementary Table S1). KBS was also used to calculate the total intake of energy (kJ/day) and macronutrients (g/day). To account for differences in energy intake across the population, the food intake for each participant was divided by their individual energy intake and multiplied by the mean energy intake of the population. Subsequently, each food variable was standardized to have a zero mean and a variance of one.

### 2.4 Anthropometric and metabolic measures

As part of Tromsø7, trained personnel conducted measurements of waist circumference (cm), height (cm) and weight (kg) of the participants, under conditions of light clothing and without shoes. Waist circumference was measured with a Seca measurement tape at level of the umbilicus. The Jenix DS-102 (DongSahn Jenix, Seoul, Korea) was utilized for height and weight measurements. The body mass index (BMI) was calculated as weight divided by height squared (kg/m^2^).

Blood samples were collected non-fasting. Levels of HDL-cholesterol (mmol/L), triglycerides (mmol/L), and CRP (mg/L) were analyzed using enzymatic colorimetric methods by the Cobas 8000 c702 instrument (Roche Diagnostics, Mannheim, Germany). HbA1c (%) was analyzed by high-performance liquid chromatography using Tosoh G8 instrument (Tosoh Bioscience, San Francisco, USA). The blood pressure (mmHg) of the participants was measured three times using an automatic oscillometric digital device (Dinamap ProCare 300 monitor, GE Healthcare, Norway), recording the average of the last two measurements.

### 2.5 Lifestyle and demographic variables

In studying associations between diet and health, it is important to adjust for confounders. In addition to age, the given survey data included self-reported education level, physical activity level, and smoking status. The participants also reported on mean weekly alcohol consumption (dl/day) as part of the FFQ. This variable is retained as a continuous variable, avoiding potential loss of information due to categorization (21) and arbitrary cut-off points (22).

The variable on education level included four categories: Primary/partly secondary education, upper secondary education, short tertiary education, and long tertiary education. Physical activity level was assessed using the four-level activity scale developed by Saltin-Grimby (23), including sedentary (mainly reading/watching TV), light (walking/biking more than 4 h/week), moderate (exercise more than 4 h/week), and vigorous activity level (hard exercise/competitive sports more than 4 h/week). Due to the infrequent reporting of vigorous activity, this category was merged with moderate activity and labeled as high activity level. Smoking status was categorized as smoker or non-smoker, the latter category also including previous smokers.

### 2.6 Statistical method

**Figure 1:**
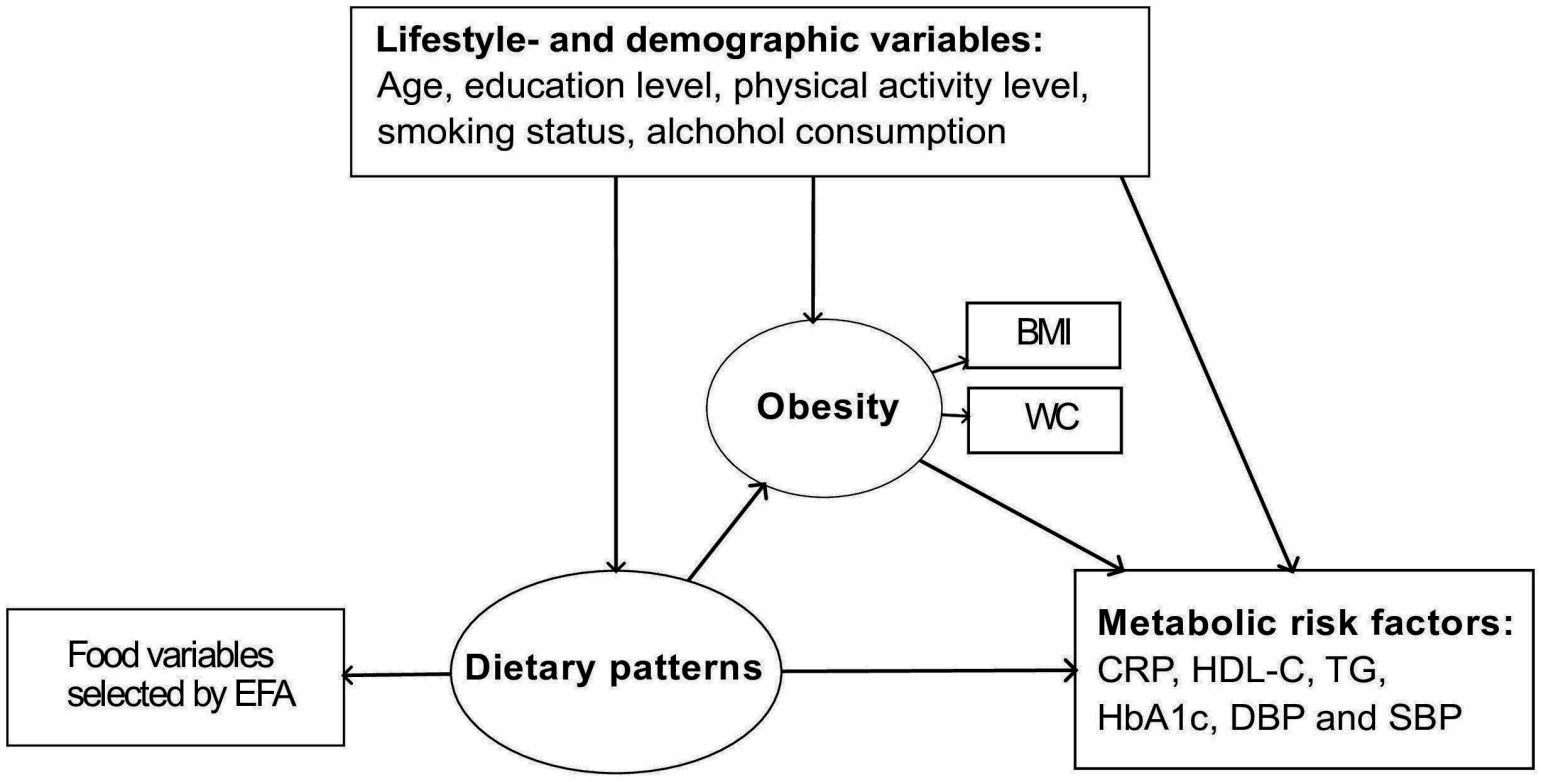
Simplified path diagram illustrating the assumed theoretical structure of the model. Circles represent latent variables, while squares represent observed values. BMI, body mass index; WC, waist circumference; CRP, C-reactive protein; HDL-C, high density lipoprotein cholesterol; TG, triglycerides; HbA1c, hemoglobin A1c; DBP, diastolic blood pressure; SBP, systolic blood pressure.

The primary focus of this paper was to explore the associations between dietary patterns and established metabolic risk factors for cardiovascular disease utilizing the framework of ESEM. All analyses were performed separately for women and men. A simplified representation of the ESEM is depicted in Figure 1.

In order to test different ESEMs, we initially used a separate EFA with varimax rotation for the 35 aggregated food variables. This was performed as an initial variable selection step to reduce model complexity, as inclusion of weakly correlated variables in ESEM might introduce unnecessary noise and give poor performance. Specifically, we chose to discard food variables having an absolute value for the loadings less than 0.3. The cuf-offs for the number of factors for this EFA was determined based on the scree plot of eigenvalues in which the localization of the so-called “elbow” indicated where the eigenvalues leveled off. Results incorporating all food variables in the ESEM are provided in the Supplementary Section 5.

The ESEM was fitted assuming a predefined number of underlying dietary patterns. The dietary patterns represent latent overlapping factors, constructed to explain the correlation structure of the observed food variables. The dietary patterns are modeled to have a direct effect on both obesity and the six metabolic risk factors: HDL-cholesterol, triglycerides, CRP, HbA1c, and systolic- and diastolic blood pressure. The values of HDL-cholesterol, triglycerides, and CRP were log-transformed, as these measures had highly left skewed distributions. The log-transformation gives distributions that are closer to normal and reduces variance. Also, this transformation emphasizes differences at low values. This is advantageous, especially for CRP measures, as the focus here is on elevated levels rather than high levels due to acute infection.

The ESEM includes obesity as a mediator between dietary patterns and the metabolic risk factors. Obesity is modeled as a latent variable, based on observed BMI and waist circumference. The resulting model structure isolates the direct dietary effects on the metabolic risk factors, not being influenced by obesity. Also, it captures the indirect effects as the product of the estimated regression coefficients between dietary patterns and obesity, and between obesity and the risk factors. The total effect of diet on the metabolic risk factors sums up the indirect effect from diet through obesity and the direct effect of diet. All the structural regression equations of the model were adjusted for confounding effects of lifestyle- and demographic variables, including age, education level, physical activity level, smoking status, and alcohol beverage consumption.

The unknown parameters of the resulting ESEM, including factor loadings and regression coefficients, were estimated using the maximum likelihood method. This method assumes that the observed variables have multivariate normal distributions, but it is quite robust to departures from normality in the data. The robust standard errors and the Satorra-Bentler scaling of test statistics were employed to account for discrepancy from the normality assumption. The reported estimates are not standardized and the variance of the latent variables are fixed to one. As large variation in measurement scales can cause numerical problems in fitting the ESEM, waist circumference was represented in decimeters and blood pressure in cmHg (24). *P-*values < 0.01 are considered significant.

The model fit was assessed using the comparative fit index (CFI), the root mean square error of approximation (RMSEA) and the standardized root mean squared residual (SRMR). Common cut-offs used to indicate acceptable model fit is CFI>0.90, RMSEA < 0.08 and SRMR < 0.08. Additionally, Monte Carlo simulated tailored cut-offs were computed.

## 3 Results

### 3.1 Characteristics of the study sample

The study sample included a total of 5365 women (53.7%)and 4623 men aged 40 to 79 years. The mean age of the women was 55.7 years, being slightly lower than the mean age of 57.0 years for the men. More than half of the participants had tertiary education, the proportion being 55.9% among women and 52.6% among men. About 12% of both women and men reported to have a sedentary physical activity level, while the majority reported light physical activity level. More men than women reported a high physical activity level, the percentages being 36.1% among men vs. 22.9% among women. Approximately 12% of the participants were smokers. The energy adjusted median alcohol consumption was 0.69 dl/day for women and 1.41 dl/day for men, see Supplementary Table S2 for more details.

### 3.2 Initial food variable selection using EFA

A selection of the 35 food variables was determined based on EFA with four factors, retaining food variables with absolute loadings higher than 0.3. In the subsequent analysis using the main ESEMs, this implied that the latent dietary patterns were constructed based on a selection of 21 food variables for women and 18 food variables for men.

### 3.3 Characteristics of dietary patterns estimated by ESEM

The main ESEMs for both women and men were fitted using the selected food variables by EFA and four dietary patterns.

The estimated loadings for the four factors are displayed in Table 1. These reflect three quite similar dietary patterns for women and men. The first factor (diet 1) represents a Snacks and Meat pattern, having the highest positive loadings on candy, chips, cakes, and pastries, compound meat dishes, and in addition rice and pasta for women and processed meat for men. In contrast, this pattern showed negative loadings on unprocessed fish. The second dietary pattern (diet 2) is referred to as a Health-conscious pattern. It showed high positive loadings on unprocessed fish, Asian dishes, chicken, and vegetables but negative loadings on bread. The third common dietary pattern is seen as a Processed Dinner pattern, characterized by high positive loadings on processed fish and meat, red meat, potato, and sauce. For women, the fourth factor is interpreted as a Porridge pattern (diet 4a), showing the highest positive loadings on sweetened porridge (rice and sour cream porridge), pancakes, and sweetened breakfast cereals. The fourth factor for men is referred to as a Cake pattern (diet 4b), having the highest positive loading on cakes and pastries but also a high positive loading on compound meat dishes.

**Table 1:** Dietary factor loadings estimated by ESEM.

	**Women**				**Men**			
**Food variables**	**Diet 1**	**Diet 2**	**Diet 3**	**Diet 4a**	**Diet 1**	**Diet 2**	**Diet 3**	**Diet 4b**
Candy	0.26	-0.09	0.00	0.01	0.18	-0.02	0.01	0.04
Chips	0.28	-0.07	0.02	-0.11	0.25	0.03	0.03	-0.02
Cakes/Pastries	0.32	0.04	0.06	0.25	0.18	0.12	0.01	0.65
Compound meat dishes	0.45	0.06	0.12	0.12	0.38	0.20	0.07	0.48
Rice/Pasta	0.28	0.12	0.03	-0.01	0.12	0.29	-0.04	0.02
Fish, unprocessed	-0.30	0.41	0.15	-0.11	-0.34	0.29	0.21	-0.07
Asian dishes	0.01	0.34	-0.11	0.05	-0.04	0.28	0.04	-0.02
Chicken	0.15	0.44	0.05	-0.20	0.01	0.35	0.02	0.00
Vegetables	-0.17	0.57	-0.04	-0.20	-0.19	0.36	0.05	-0.06
Fish, processed	-0.04	0.11	0.34	0.09	-0.09	0.07	0.28	0.06
Meat, processed	0.12	-0.14	0.41	0.06	0.25	-0.16	0.47	0.05
Meat, red	0.02	0.12	0.48	-0.04	0.06	0.11	0.46	-0.05
Potato	-0.11	-0.13	0.36	0.05	-0.11	-0.09	0.38	-0.03
Sauce etc.	0.14	0.09	0.33	-0.05	0.10	0.18	0.34	-0.01
Porridge/Pancakes	0.06	-0.08	0.13	0.47	0.00	-0.10	0.07	0.27
Breakfast cereals, sweetened	0.01	-0.04	-0.02	0.40	-0.07	-0.03	-0.08	0.17
Bread	-0.08	-0.39	-0.05	-0.09	-0.04	-0.30	-0.26	-0.12
Meat spread	0.09	-0.14	0.05	-0.22	0.13	-0.13	-0.12	-0.12
Fish spread	-0.16	0.06	-0.02	-0.21				
Nuts	-0.09	0.19	-0.32	-0.01				
Breakfast cereals/Porridge, unsweetened	-0.07	0.22	-0.22	0.15				

Diet 1: Snacks and Meat pattern, Diet 2: Health-conscious pattern, Diet 3: Processed Dinner pattern, Diet 4a: Porridge pattern, Diet 4b: Cake pattern.

Table 2 displays the estimated coefficients of lifestyle and demographic variables in the regression models using each of the dietary pattern as the dependent variable. For both sexes, higher age was negatively associated with Snacks and Meat and the Health-conscious patterns, while being positively associated with the Processed Dinner pattern. Further similarities in the analysis for women and men include a positive association between higher education level and the Health-conscious pattern and a negative association with the Processed Dinner pattern. Compared to a sedentary activity level, light and high activity levels were negatively associated with the Snacks and Meat and the Processed Dinner patterns, while a high activity level was positively associated with the Health-conscious pattern for both sexes. Smoking was positively associated with the Processed Dinner pattern and negatively associated with the Health-conscious pattern. Smoking and alcohol consumption were negatively associated with the Porridge pattern for women and the Cake pattern for men.

**Table 2:** Estimated regression coefficients of lifestyle and demographic variables in explaining each dietary pattern (dependent variable).

	**Women**				**Men**			
**Confounders**	**Diet 1**	**Diet 2**	**Diet 3**	**Diet 4a**	**Diet 1**	**Diet 2**	**Diet 3**	**Diet 4b**
Age	-0.116^*^	-0.013^*^	0.024^*^	0.017^*^	-0.123^*^	-0.056^*^	0.043^*^	0.009
Lower secondary (ref)	0	0	0	0	0	0	0	0
Upper secondary	0.05	0.25^*^	-0.31^*^	-0.16	0.21^*^	0.40^*^	-0.26^*^	-0.07
Short tertiary	0.19^*^	0.33^*^	-0.57^*^	-0.14	0.16	0.63^*^	-0.51^*^	-0.10
Long tertiary	0.13	0.51^*^	-0.83^*^	-0.06	0.05	0.92^*^	-0.94^*^	0.05
Physical activity level:								
Sedentary (ref)	0	0	0	0	0	0	0	0
Light	-0.27^*^	0.30^*^	-0.37^*^	0.02	-0.41^*^	0.12	-0.30^*^	0.11
High	-0.20^*^	0.58^*^	-0.64^*^	0.04	-0.53^*^	0.25^*^	-0.49^*^	0.14
Smoking status:								
Non-smokers (ref)	0	0	0	0	0	0	0	0
Smokers	-0.02	-0.32^*^	0.42^*^	-0.29^*^	0.11	-0.25^*^	0.26^*^	-0.23^*^
Alcohol consumption	-0.07	0.08	0.05	-0.34^*^	0.11^*^	0.10^*^	0.04	-0.18^*^

Diet 1: Snacks and Meat pattern, Diet 2: Health-conscious pattern, Diet 3: Processed Dinner pattern, Diet 4a: Porridge pattern, Diet 4b: Cake pattern. *p < 0.01. Lower secondary education category includes primary/partly secondary level.

### 3.4 Direct, indirect and total effects of dietary patterns estimated by ESEM

**Figure 2:**
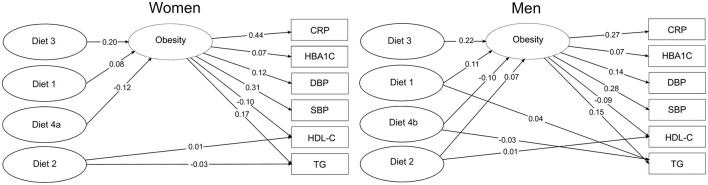
Significant effects between dietary patterns and metabolic CVD risk factors including obesity as a mediator. Diet 1: Snacks and Meat pattern, Diet 2: Health-conscious pattern, Diet 3: Processed Dinner pattern, Diet 4a: Porridge pattern, Diet 4b: Cake pattern.

All significant associations between dietary patterns, obesity, and metabolic risk factors are displayed in Figure 2. These include direct effects between dietary patterns on the metabolic risk factors, and also the effects through the latent obesity variable. All of the underlying regression models were adjusted for the confounding effects of the lifestyle and demographic variables given in Section 2.5.

The Health-conscious pattern showed a direct positive effect on HDL-cholesterol and a direct negative effect on triglycerides (women only). The Snacks and Meat pattern for men was estimated to have an unfavorable direct effect on triglycerides, while the Cake pattern showed a favorable direct effect, decreasing the level of triglycerides.

All the dietary patterns were seen to be directly associated with obesity, except for the Health-conscious pattern for women. Specifically, the Snacks and Meat, the Health-conscious (men only) and the Processed Dinner patterns showed significant positive association with obesity, while the Porridge and Cake patterns had a negative effect. Obesity was consistently found to have a significant adverse effect on all of the given metabolic risk factors, increasing the levels of CRP, HbA1c, triglycerides, and blood pressure, while decreasing the level of HDL-cholesterol.

The complete mediation analysis of direct, indirect and total effect is summarized in Table 3. All of the estimated indirect effects were seen to be significant, except for the Health-conscious dietary pattern in women. Specifically, the indirect effects of the Snacks and Meat pattern, the Health-conscious pattern (men only) and the Processed Dinner pattern showed unfavorable effects on the metabolic risk factors, while the Porridge pattern and the Cake pattern had favorable effects.

**Table 3:** Direct, indirect and total effect of diet and obesity on metabolic CVD risk factors.

**Women**	**Effects**	**Obesity**	**CRP**	**HDL-C**	**TG**	**HbA1c**	**SBP**	**DBP**
Diet 1	Direct	0.08^*^	0.02	-0.01	-0.01	-0.01	0.00	0.00
	Indirect		0.04^*^	-0.01^*^	0.01^*^	0.01^*^	0.03^*^	0.01^*^
	Total		0.06^*^	-0.02^*^	0.00	-0.01	0.02	0.01
Diet 2	Direct	0.02	-0.03	0.01^*^	-0.03^*^	-0.01	-0.06	0.00
	Indirect		0.01	0.00	0.00	0.00	0.00	0.00
	Total		-0.03	0.01	-0.03^*^	-0.01	-0.05	0.00
Diet 3	Direct	0.20^*^	0.02	0.00	0.02	-0.01	0.03	0.00
	Indirect		0.09^*^	-0.02^*^	0.03^*^	0.01^*^	0.06^*^	0.02^*^
	Total		0.11^*^	-0.02^*^	0.05^*^	0.01	0.09^*^	0.02
Diet 4a	Direct	-0.12^*^	0.00	-0.01	0.00	0.01	-0.03	-0.02
	Indirect		-0.05^*^	0.01^*^	-0.02^*^	-0.01^*^	-0.04^*^	-0.01^*^
	Total		-0.05^*^	0.00	-0.02	0.00	-0.06	-0.04
**Men**	**Effects**	**Obesity**	**CRP**	**HDL-C**	**TG**	**HbA1c**	**SBP**	**DBP**
Diet 1	Direct	0.11^*^	0.05	-0.02	0.04^*^	-0.01	0.04	0.03
	Indirect		0.03^*^	-0.01^*^	0.02^*^	0.01^*^	0.03^*^	0.02^*^
	Total		0.08^*^	-0.03^*^	0.06^*^	0.00	0.07	0.05^*^
Diet 2	Direct	0.07^*^	0.02	0.01^*^	-0.02	-0.02	0.01	0.00
	Indirect		0.02^*^	-0.01^*^	0.01^*^	0.00^*^	0.02^*^	0.01^*^
	Total		0.04	0.01	-0.01	-0.02	0.02	0.01
Diet 3	Direct	0.22^*^	0.01	0.01	-0.01	0.00	0.00	0.02
	Indirect		0.06^*^	-0.02^*^	0.03^*^	0.01^*^	0.06^*^	0.03^*^
	Total		0.06^*^	-0.02^*^	0.02	0.01	0.06	0.05
Diet 4b	Direct	-0.10^*^	-0.03	0.01	-0.03^*^	-0.01	0.03	-0.01
	Indirect		-0.03^*^	0.01^*^	-0.02^*^	-0.01^*^	-0.03^*^	-0.01^*^
	Total		-0.06^*^	0.02^*^	-0.04^*^	-0.02	0.01	-0.02

CRP, C-reactive protein; HDL-C, HDL-cholesterol; TG, triglycerides; SBP, systolic blood pressure; DBP, diastolic blood pressure. ^*^P-value < 0.01.

For both sexes, the Snacks and Meat pattern and the Processed Dinner pattern were estimated to have an unfavorable total effect on CRP and HDL-cholesterol. The Porridge pattern and the Cake pattern were seen to have a favorable total effect on CRP. For men, the Snacks and Meat pattern had a total unfavorable effect on triglycerides and diastolic blood pressure and the Cake pattern had a favorable total effect on the levels of HDL-cholesterol and triglycerides. The Health-conscious pattern for women showed a favorable total effect on the levels of triglycerides, retaining the favorable direct effect. However, the total effect of the Health-conscious pattern on HDL-cholesterol was not significant for neither women nor men, despite the favorable direct effect. For men, obesity seemed to act as a competitive mediator as the direct effect was positive while the indirect effect was negative giving a non-significant total effect. In women, we also noticed unfavorable total effects of the Processed Dinner pattern on triglycerides and systolic blood pressure.

### 3.5 Estimated effects of confounders on obesity and metabolic risk factors

Age was positively associated with all metabolic risk factors among women (Supplementary Table S3). Among men, age was positively associated with obesity and with all risk factors except for triglycerides and diastolic blood pressure. Significant associations seen for both women and men include a negative association between long tertiary education level and obesity. Further, light and high activity levels were negatively associated with obesity and a high activity level was associated with lower CRP and higher HDL-cholesterol levels. Smokers showed lower rates of obesity, lower HDL-cholesterol, and higher levels of CRP, triglycerides and HbA1c. Higher consumption of alcoholic beverage was associated with increased HDL-cholesterol levels and diastolic blood pressure. For a detailed overview of all estimated effects of confounders, see Supplementary Table S3.

### 3.6 Evaluation of the model

To explore alternatives to the main ESEMs, models with three dietary patterns were fitted using both a selection of the aggregated food variables found by EFA and all of them. In addition, models with four dietary patterns and all food variables were fitted (Supplementary Section 5).

The common dietary patterns (Diet 1–3), were identified consistently across all models fitted. However, the main models using four dietary patterns and a selection of the food variables were the only ones giving CFI >0.90. All of the fitted models clearly met the cut-offs of RMSEA < 0.08 and SRMR < 0.08 (Supplementary Table S16). Fitting the main ESEM for women, the goodness-of-fit indexes were CFI = 0.908, RMSE = 0.039, and SRMR = 0.025. For men, the corresponding measures were CFI = 0.917, RMSE = 0.041, and SRMR = 0.026. Using the alternative models, both RMSEA and SRMR increased slightly (Supplementary Table S16).

The goodness of fit indexes can improve by fitting models with a higher number of dietary patterns, but this also increases model complexity. Here, the main models using four patterns and a selection of the food variables are seen to represent a reasonable trade-off between goodness-of-fit, interpretation and simplicity. Simulated data with a similar correlation structure and number of observations, but with normal distributions, resulted in tailored cut-offs around CFI >0.998, RMSE < 0.006, and SRMR < 0.011.

## 4 Discussion

### 4.1 Dietary patterns and metabolic risk factors for CVD

Four dietary patterns and their effects on metabolic risk factors have been investigated utilizing ESEM. The study sample is assumed to represent a general Nordic population and the large sample size enabled separate analysis for women and men. The analysis provided estimates of direct, indirect and total effects of dietary patterns on metabolic CVD risk factors, modeling obesity as a mediator. The three patterns labeled the Snacks and Meat pattern, the Health-conscious pattern and the Processed Dinner pattern, were similar for women and men. In addition, the analysis identified a sweetened Porridge pattern for women and a Cake pattern for men.

The Snacks and Meat pattern shares similarities with dietary patterns previously labeled as Western, Sweets and Snacks, Unhealthy, Modern, Fast Food, or Convenience-food (25). The Health-conscious pattern shares similarities with dietary patterns previously labeled as Healthy, Southern or Prudent, and also resembles a Mediterranean diet. This pattern was associated with a healthier lifestyle, characterized by increased physical activity levels and less smoking, indicating that it is commonly followed by health-conscious participants. The Processed Dinner pattern resembles a more traditional Norwegian dietary pattern having high loadings on fish product variables and potatoes. However, this pattern was also rich on meat and processed food and appeared to be associated with a less healthy lifestyle, such as smoking and lower physical activity levels. Both the Porridge pattern (women) and the Cake pattern (men) were associated with lower alcohol consumption.

In general, vegetables and minimally processed meat and plants have consistently shown associations with better health in several analyses (26, 27). This was also seen in our analysis. By assuming obesity to act as a mediator between dietary patterns and metabolic risk factors, the direct effect of diet on the risk factors is isolated. We observed direct favorable effects of the Health-conscious pattern on HDL-cholesterol (both women and men) and triglycerides (only women). This is in coherence with previous studies adjusting for BMI, having linked dietary patterns rich in vegetables, fruits, whole grains and fish with lower triglyceride levels (10, 28–30), higher HDL-cholesterol levels (30–32), and lower blood glucose levels (29, 30, 33). Favorable effects of healthy diets on HDL-cholesterol and triglycerides have also been found in randomized controlled trials (34).

Dietary patterns characterized by high consumption of meat and processed foods are associated with unfavorable trends in metabolic risk factors. This includes negative association with HDL-cholesterol (11, 31, 33, 35, 36) and positive association on fasting blood glucose (33, 35, 36). Among men, our study showed a direct unfavorable effect of the Snacks and Meat pattern on triglycerides.

Surprisingly, the Cake pattern was seen to have a favorable association with triglyceride levels among men. This pattern was ambiguous, giving a high loading on cake but also on compound meat dishes. Dietary patterns including sweets have been reported to not have an association with metabolic risk factors (29, 31, 37). Other studies have in fact found health-favorable associations between cake/sugar and metabolic risk factors, also suggesting various explanations (38, 39). One such possible explanation is under-reporting of unhealthy food choices (40, 41). An alternative explanation could be that individuals who are overweight or have lifestyle-related diseases, may adopt changes in lifestyle behavior like dieting. This is commonly motivated by reasons such as weight management or addressing health problems (42, 43). Both the Cake pattern and the Porridge pattern were negatively associated with obesity. This might seem counter-intuitive but is supported by previous findings. For instance, free sugar intakes from cakes, pies, and biscuits for men, and breakfast cereals for women, have been found to reduce the probability of obesity (39).

Obesity is an important risk factor for cardiovascular disease. This emphasizes the necessity of promoting healthy dietary choices to a population to reduce obesity rates. Various analyses have observed the inverse relationship between obesity and dietary patterns rich in vegetables, fruits, and nuts, combined with low intake of meat and sweets (44). Both the Snacks and Meat pattern and the Processed Dinner pattern showed positive associations with obesity for both sexes. This association resulted in an overall unfavorable effect on several metabolic CVD risk factors. Although the Health-conscious pattern was positively associated with obesity in men, its direct effect on HDL-cholesterol was favorable. For women, the Health-conscious pattern was not significantly associated with obesity, possible due to small differences in these patterns between men and women. Given the importance of obesity in explaining the risk factors, this typically gave unfavorable indirect effects of Diet 2 for men, while giving non-significant indirect effects for women.

The estimated direct effects largely align with the indirect effects mediated by obesity. Diets associated with one aspect of health have been seen to influence other health aspects as well (26). In our analysis, diets rich in vegetables, fish, and low unprocessed foods are preferable for mitigating several risk factors for cardiovascular disease, compared to diets high in processed foods and sweets. This finding is consistent with existing literature and dietary recommendations (26).

### 4.2 Model fit

The main ESEM was deemed a useful representation of the main known effects of dietary pattern on recognized CVD risk factors. The fit measures of the model were comparable to similar analysis and were within traditionally accepted cut-offs (14, 45). Measures of model fit can be sensitive to factors beyond model misspecification. Therefore, the traditional practice of applying fixed cut-offs, independent of research areas, can be problematic (46, 47). To investigate this further, we simulated tailored cut-offs based on a model with structure similar to the real data. This led to more stringent criteria, none of which were met by any of the tested models.

The model fit criteria for the ESEM can be improved by increasing the number of dietary patterns used. However, this might produce instability in the factor structure and undermine interpretability of the patterns. The use of four factors in the current analysis was considered to be within limits of interpretation. Alternatively, one could argue for using three factors, being in coherence with the number of patterns previously found by cluster analysis (48). The supplementary analysis using three factors in the ESEM supported identification of the three main common dietary patterns for men and women, but the CFI then dropped below the traditionally recommended cut-off.

### 4.3 Model specification

The presented models assume that dietary behavior affect obesity, as measured by waist circumference and BMI. This assumption is common (14, 49). However, obesity and lifestyle-related illness can also influence dietary behavior and this is the reason why participants with diabetes were excluded from our analysis. This issue also extends beyond diabetic patients, as the relation between an unhealthy diet and overweight is common knowledge. It is reasonable to assume that some participants have made dietary changes to reduce their weight. However, if all other assumptions hold, the analysis of direct effects should remain consistent.

### 4.4 Strengths and limitations

The given study was conducted on a large dataset, allowing separate analysis for women and men. The results were consistent across genders, which is considered a strength. All regression equations were adjusted to account for the confounding effect of age. However, further analysis is needed to fully explore consistency of the estimated effects across different age groups, such as by stratifying participants into 10-year age intervals. Given the data-driven approach, the identified dietary patterns will have varying degrees of dissimilarities across age groups, and estimated effects cannot be interpreted in an unambiguous way. Additionally, statistical power decreases with smaller sample sizes, which typically reduces the number of significant findings. However, preliminary analyses have consistently identified a Processed dinner pattern in all age groups, having a significant positive association with obesity. Also, obesity has been seen to have a consistent significant adverse effect on all the given metabolic risk factors, independent of age.

The flexible framework of ESEM enabled estimation of all factor loadings and associations simultaneously, also adjusting for several lifestyle and demographic variables. Measurements of the metabolic risk factors were available on a continuous scale which is important to retain information (21, 22). To avoid inflated probabilities of false positive findings due to multiple testing, we chose a quite conservative significance level of 0.01 for all hypotheses tests. This was seen to be more conservative than using a global significance level of 0.05 and making adjustments according to the method of false discovery rate (50).

The cross-sectional design imposes limitations on the interpretations and strengths of the model assumptions. Our analysis rely on theoretical foundations and updated understanding of the associations between variables. However, interpretation of results as causal relationships must be done with care due to the cross-sectional design. The observed associations can have multiple explanations and the tested models are constrained to assuming linear relationships between variables. Also, self-reported dietary data could be prone to recall bias and under-reporting, especially for food items perceived as unhealthy.

### 4.5 Potential of ESEM in nutritional research

ESEM offers a comprehensive modeling framework for simultaneously deriving dietary patterns and estimating their associations with health risk factors. The EFA component allows for estimation of cross-loadings, reflecting that food variables can contribute to multiple dietary patterns. The SEM component helps to disentangle the potentially intricate relationships between dietary patterns and risk factors. By incorporating mediation analysis, SEM facilitates joint estimation of direct and indirect effects, allowing for a more precise description of dietary effects on health outcomes than using traditional regression models. Furthermore, the use of path diagrams effectively visualizes the underlying assumptions of the model and the chain of reasoning. Different model formulations and pathways can be tested and compared using global model fit indices and the model accounts for measurement error in dietary patterns and other latent variables.

Although ESEM was introduced in nutritional research almost a decade ago (14), its use remains rather limited. The two steps of deriving dietary patterns and estimating their relationships with health outcomes are commonly performed separately instead of jointly. As a first step, dietary patterns are often derived using exploratory and/or confirmatory analysis. A separate second step is then implemented to estimate associations with health outcome variables, often using multiple regression models or SEM. SEM is more restrictive than ESEM as cross-loadings are usually fixed to zero. However, in studying health outcomes for well-defined dietary patterns, the use of SEM rather than ESEM could simplify model interpretation. SEM has been used to study the direct and indirect effects of dietary patterns on a number of health outcome variables like hypertension (45), obesity (49, 51), elevated blood glucose level (52), and homocysteine level (53). Commonly, several different pathways can be considered, and dietary patterns could also be of use as mediators (54).

A great advantage of SEM and ESEM compared to traditional regression models is the simultaneous assessment of all associations and assumed pathways. However, this poses the question on correction for multiple testing to avoid increased risk of Type I errors and false positive findings. Both SEM and ESEM are commonly evaluated only using global fit indices like CFI and RMSEA. Although these fit measures are within traditionally accepted cut-offs, this does not necessarily prevent inflated type I error (55). A recommendation for future studies could be to also consider adjustments of p-values due to multiple testing. For example, this can be done according to the method of false discovery rate (50, 55), being less strict than a Bonferroni correction.

### 4.6 Conclusion

The given study used ESEM to simultaneously identify dietary patterns and estimate their impact on metabolic CVD risk factors, mediated by obesity. Separate analysis for women and men supported the identification of three common and stable dietary patterns labeled Snacks and Meat, Health-conscious and Processed Dinner. In addition, a fourth factor identified a sweetened Porridge pattern for women and a Cake pattern for men. However, these two patterns were less stable across models and should be interpreted with care.

Except for the Health-conscious pattern among women, all dietary patterns were significantly associated with obesity and showed indirect effects on all metabolic risk factors. This highlights the importance of adjusting for obesity, such as including it as a mediator, when investigating the direct effect of dietary patterns on metabolic risk factors. The current study demonstrated a favorable direct effect of the Health-conscious pattern on blood lipid levels, like HDL-cholesterol and triglycerides (only women), despite a positive association with obesity for men. Additionally, the Snacks and Meat pattern was found to have an unfavorable direct effect on triglycerides for men.

## Data Availability

The data analyzed in this study is subject to the following licenses/restrictions: the dataset is obtained from a third party (the Tromsø Study) and is not publicly available. Requests to access these datasets should be directed to the Tromsø Study (https://uit.no/research/tromsostudy).
