# Supplementary material for: Analysis of dietary pattern effects on metabolic risk factors using structural equation modeling
Source: Front Nutr. 2025 Jun 25;12:1540919. doi: 10.3389/fnut.2025.1540919 (PMC12237654; doi:10.3389/fnut.2025.1540919)
Supplement: Supplementary file 1 [file Data_Sheet_1.pdf]

Supplementary Material

1 INCLUSION/EXCLUSION CRITERIA

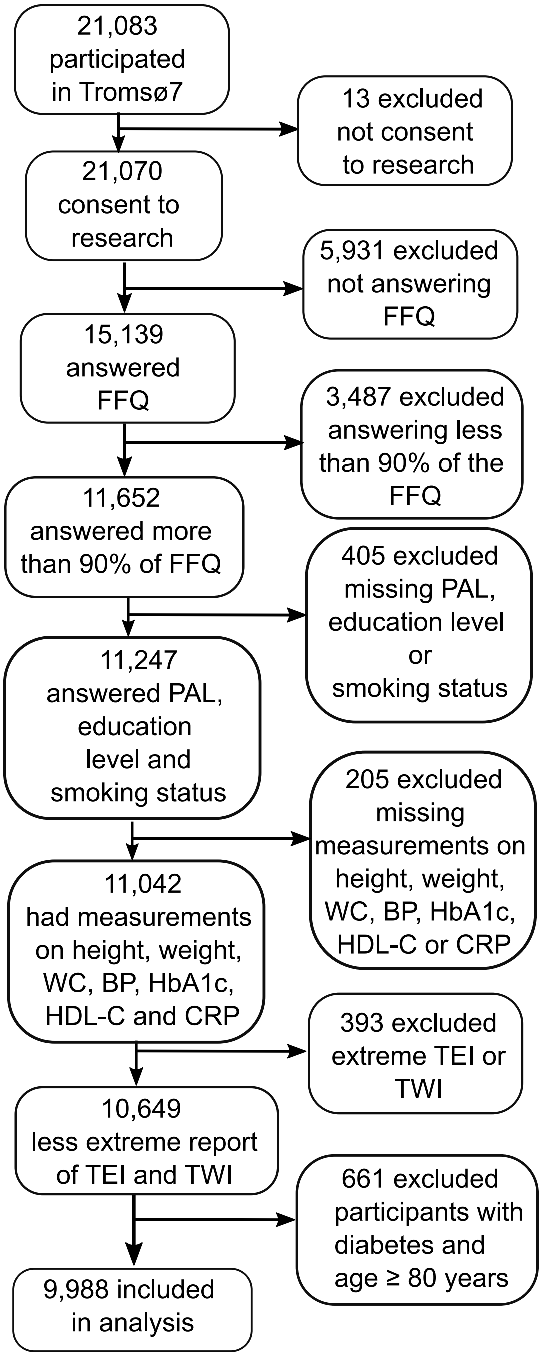

Figure S1: Flow diagram illustrating the inclusion/exclusion criteria for participants in the study. FFQ: food frequency questionnaire, PAL: Physical activity level, WC: waist circumference, BP: blood pressure, HbA1c: glycated hemoglobin, HDL-C: HDL-cholesterol, CRP: C-reactive protein, TEI: Total energy intake, TWI: Total water intake

2 AGGREGATED FOOD VARIABLES

Variables from the food frequency questionnaire (FFQ) were originally aggregated into 39 food variables. In our analysis, the water and tea variables were excluded due to low correlation with all of the other aggregated variables. We also excluded all variables on supplements. The aggregated variable on Beverages with alcohol was included as a lifestyle variable, rather than a food variable.

Table S1: Aggregated food variables based on the 261 questions from the FFQ

Variable name	Food variables from FFQ
Bread	BREAD_WHITE, BREAD_WHOLEGRAIN50, BREAD_WHOLEGRAIN100, CRISPBREAD_WHITE, CRISPBREAD_WHOLEGRAIN
Butter and Margarine	BUTTER, MARGARINE_BREMYKT, MARGARINE_BRELETT, MARGARINE_SOFT_SFSE, MARGARINE_VITA, MARGARINE_SFV_LIGHT, MARGARINE_MELANGE, MARGARINE_OTHER
Mayonnaise and Plant-based Oils	OIL_OLIVE_OTHER, MAYONNAISE, MAYONNAISE_SALAD, MAYONNAISE_SAL_LIGHT
Cheese	CHEESE_WHEY, CHEESE_WHEY_LIGHT, CHEESE_WHITE, CHEESE_WHITE_LIGHT, CHEESE_BLUE_DESSERT, CHEESE_SOFT, CHEESE_SOFT_LIGHT, COTTAGECHEESE
Meat spread	LIVERPASTE, LIVERPASTE_LIGHT, SERVELAT, HAM_BOILED, SALAMI
Fish spread	CAVIARSPREAD, CAVIARSPREAD_SVOLVAR, MACKEREL_TOMATOSAUCE, SALMON_TROUT_SMOKED, SARDINES_HERRING, TUNA, SCHRIMP_CRAB
Egg	EGG
Jam	JAM_MARMELADE, JAM_LIGHT, PEANUTBUTTER, CHOCOLATE_NUT_SPREAD, SWEET_SPREAD
Breakfast cereals/Porridge, unsweetened	PORRIDGE_OATMEAL, OATMEAL_4GRAIN, CEREAL_UNSWEETENED, CEREAL_ALLBRAN
Breakfast cereals, sweetened	CEREAL_SWEETENED, CORNFLAKES, CEREAL_HONEY, CEREAL_PUFFED_RICE_OAT, JAM_CEREAL, SUGAR_CEREAL
Milk	MILK_WHOLE, MILK_SEMISKIMMED, MILK_EXTRASEMISKIMMED, MILK_SKIMMED, MILK_BIOLA_CULTURA_NA, MILK_BIOLA_CULTURA_FL, MILK_FL, HOTCHOCOLATE

Yoghurt	YOGHURT_DRINK, YOGHURT_NATURAL, YOGHURT_FRUIT, YOGHURT_GOMORGEN_MUSLI, YOGHURT_LIGHT_FRUIT, YOGHURT_LIGHT_MUSLI
Water	WATER_TAP, WATER_BOTTLE
Juice	JUICE_ORANGE, JUICE_APPLE_OTHER, NECTAR_APPLE_OTHER
Soft drinks	SAFT_SUGAR, SAFT_ARTIFICIAL, SOFTDRINK_SUGAR, SOFTDRINK_ARTIFICIAL, ICETEA_SUGAR, ICETEA_ARTIFICIAL, BEER_NONALCOHOLIC
Beverages with alcohol	BEER_STRONG_PILS, BEER_LIGHT, CIDER_ALCOPOPS, WINE_RED, WINE_WHITE, WINE_FORTIFIED, LIQUOR, COCKTAIL
Coffee	COFFEE_BOILED, COFFEE_FILTERED, COFFEE_INSTANT, COFFEE_ESPRESSO, COFFEE_LATTE, COFFEE_CAPPUCINO
Tea	TEA_BLACK, TEA_GREEN, TEA_HERBS
Meat, processed	SAUSAGE_REDMEAT, SAUSAGE_REDMEAT_LIGHT, SAUSAGE_CHICKEN_TURKEY, SAUSAGE_HOTDOG_PORK, SAUSAGE_HOTDOG_CHICKEN, HAMBURGER_WITH_BUN, KARBONADEBURGER, MEATBALL_MEATLOAF, BACON
Meat, red	STEW_MINCED_MEAT, STEAK_PORK_BEEF_LAMB, CHOPS_PORK_BEEF_LAMB, ROAST_PORK_BEEF_LAMB, ROAST_GAMEMEAT, STEW_MEAT, STEW_MEAT_LAPSKAUS
Chicken	CHICKEN_GRILLED, CHICKEN_FILLET, WOK, STEW_CHICKEN
Compound meat dishes	TACO_SHELLS_MEAT_SALAD, WRAP_TORTILLA, KEBAB, PIZZA, CALZONA, LASAGNA_MOUSAKKA
Asian dishes	PIE_QUICHE, SPRINGROLLS, SOUP_VEGETABLE, DISH_VEGETARIAN, NUDLES_INSTANT, OMELETTE
Porridge/Pancakes	PORRIDGE_SOURCREAM, PORRIDGE_RICE_MILK, PANCAKES
Fish, processed	FISH_BURGER_PUDDING, FISH_BALLS, FISH_STICKS, FISH_BAKED_GRATIN
Fish, unprocessed	FISH_LEAN_BOILED, FISH_LEAN_FRIED, HERRING_FRESH_SMOKED, MACKEREL_FRESH_SMOKED, SALMON_TROUT, STEW_SOUP_FISH, WOK_SEAVEGETABLES, SCHRIMP_CRAB

Potato	FISH_BURGER_PUDDING, FISH_BALLS, FISH_LEAN_BOILED, FISH_LEAN_FRIED, FISH_STICKS, HERRING_FRESH_SMOKED, MACKEREL_FRESH_SMOKED, SALMON_TROUT, STEW_SOUP_FISH, FISH_BAKED_GRATIN, WOK_SEAVEGETABLES, SCHRIMP_CRAB
Rice/Pasta	RICE, PASTA, HOTDOGBUN_POTATOWRAP
Vegetables	CARROT, CABBAGE, RUTABAGA, CAULIFLOWER, BROCCOLI, BRUSSELSSPROUT, ONION, LETTUCE, BELLPEPPER, AVOCADO, TOMATO, CORN, VEGETABLES_MIX_FROZEN, SALAD_MIX, BEANS_LENTILS, VEGETABLES_BREAD
Sauce etc.	SAUCE_BROWN_WHITE, SAUCE_BEARNAISE, BUTTER_MARGARINE_MELT, BUTTER_HERB, MAYONNAISE_REMOULADE, MAYONNAISE_LIGHT, SOURCREAM, SOURCREAM_LIGHT, SOURCREAM_EXTRALIGHT, SALADDRESSING, SALADDRESSING_LIGHT, SALADDRESSING_OIL, SOYSAUCE, PESTO, SALSA_TOMATOSAUCE, KETCHUP, MUSTARD
Fruit	APPLE, PEAR, BANANA, ORANGE, CLEMENTINE, GRAPEFRUIT, PEACH_NECTARINE, KIWI, GRAPE, MELON, STRAWBERRY, RASPBERRY, BLUEBERRY, CLOUDBERRY, RAISIN, FRUIT_DRIED, FRUIT_B, FRUIT_HERMETIC, FRUITSALAD
Dessert	ICECREAM, ICELOLLY_SORBET, PUDDING, SAUCE_VANILLA, CREAM_WHIPPED
Cakes/Pastries	SWEET_BUN_PRETZEL, SWEET_ROLL_CUSTARD, PASTRY_DANISH, MUFFIN_CAKE_NOICING, WAFFLE, LEFSE, CAKE_CHOCOLATE_BROWNIE, CAKE_SPONGE_CREAM, BISCUIT_SWEET, TREAT_SNOWBALL
Chocolate	CHOCOLATE, CHOCOLATE_DARK, CHOCOLATE_CONFECTIONS
Candy	PASTILLES_SUGARFREE, CANDY_LICORICE_OTHER, CANDY_MIX
Chips	CHIPS_POTATOE, SNACS_SALTY
Nuts	PEANUT_CASHEW, ALMOND_HAZELNUT_WALNUT, FRUIT_NUT_MIX

Supplements	SUPPL_CODLIVEROIL, SUPPL_CODLIVEROIL_C, SUPPL_FISHOIL_OMEGA3, SUPPL_SEALOIL_CAPSULA, SUPPL_SANASOL, SUPPL_BIOVIT, SUPPL_MULTIVIT_MINERAL, SUPPL_MULTIVIT_TAB, SUPPL_IRON_SULFATE, SUPPL_IRON_HEME, SUPPL_IRON_FERROCHEL, SUPPL_IRON_FLORADIX, SUPPL_VIT_B, SUPPL_VIT_C, SUPPL_VIT_D, SUPPL_VIT_E, SUPPL_FOLATE
Milk/Sugar for Coffee/Tea	SUGAR_COFFEE, SUGAR_TEA, SWEETENERS_COFFEE_TEA, MILK_CREAM_COFFEE_TEA

3 CHARACTERISTICS OF THE STUDY SAMPLE

Table S2. Measures on lifestyle and demographic variables for the 9988 participants of the study

	Women	Men
Mean age (year)	55.7	57.0
Education level:		
Primary/partly secondary	18.5%	18.8%
Upper secondary	25.5%	28.6%
Short tertiary	19.0%	23.1%
Long tertiary	36.9%	29.5%
Physical activity level:		
Sedentary	11.7%	12.5%
Light	65.4%	51.4%
High	22.9%	36.1%
Smoking status:		
Non-smokers	86.8%	88.7%
Smokers	13.2%	11.3%
Median alcohol consumption (dl) adjusted for energy intake	0.69	1.41

4 ESTIMATED EFFECTS OF CONFOUNDERS ON OBESITY AND METABOLIC RISK FACTORS FOR THE MAIN ESEM

Table S3. Estimated regression coefficients of confounders, modelling the direct effects of dietary patterns on obesity and the direct effects of dietary patterns on the metabolic risk factors. The main ESEMs assume four dietary patterns based on a selection of the 35 aggregated food variables found by EFA.

	Obesity	CRP	HDL-C	TG	HbA1c	SBP	DBP
Women:							
Age	0.008	0.010*	0.005*	0.004*	0.011*	0.080*	0.010*
Physical activity level:							
Sedentary (ref)	0	0	0	0	0	0	0
Light	−0.50*	−0.09	0.02	0.01	0.01	0.02	−0.03
High	−0.78*	−0.18*	0.06*	−0.05	0.02	−0.06	−0.07
Education level:							
Primary/partly secondary (ref)	0	0	0	0	0	0	0
Upper secondary	0.00	0.00	0.02	0.00	−0.03	−0.17	−0.03
Short tertiary	−0.14*	−0.04	0.03	−0.04	−0.01	−0.26*	−0.05
Long tertiary	−0.21*	−0.04	0.03*	−0.03	−0.02	−0.30*	−0.07
Smoking status:							
Non-smokers (ref)	0	0	0	0	0	0	0
Smokers	−0.30*	0.17*	−0.08*	0.14*	0.12*	−0.18	−0.01
Alcohol consumption	−0.10*	0.03	0.05*	−0.01	−0.04*	0.08	0.11*
Men:							
Age	0.013*	0.016*	0.004*	−0.004	0.008*	0.047*	0.002
Physical activity level:							
Sedentary (ref)	0	0	0	0	0	0	0
Light	−0.42*	−0.11	0.00	0.03	−0.03	0.16	0.06
High	−0.74*	−0.12*	0.05*	−0.04	−0.02	0.31*	0.06
Education level:							
Primary/partly secondary (ref)	0	0	0	0	0	0	0
Upper secondary	−0.03	−0.01	−0.02	0.04	−0.03	−0.10	−0.07
Short tertiary	0.00	−0.02	−0.03*	0.04	−0.04	−0.14	−0.08
Long tertiary	−0.21*	−0.06	−0.01	0.02	−0.05*	−0.22	−0.12
Smoking status:							
Non-smokers (ref)	0	0	0	0	0	0	0
Smokers	−0.37*	0.26*	−0.07*	0.07*	0.12*	−0.22*	−0.10
Alcohol consumption	0.01	0.02	0.04*	0.00	−0.01	0.11*	0.06*

CRP: C-reactive protein, HDL-C: HDL-cholesterol, TG: triglycerides, HbA1c: glycated hemoglobin, SBP: systolic blood pressure, DBP: diastolic blood pressure.
* P-value < 0.01

5 ADDITIONAL ESEM ANALYSIS

In addition to the main ESEM analysis, three alternative models were fitted for both women and men. These include models assuming three underlying dietary patterns, both including a selection of the aggregated food variables found by EFA or all of the 35 variables. We also fitted models assuming four dietary patterns based on all of the 35 aggregated food variables. For each model, the following results are reported:

- Estimated factor loadings for the constructed dietary patterns
- The estimated regression coefficients of lifestyle and demographic variables modelling the constructed dietary patterns as dependent variables
- Figures illustrating the estimated regression coefficients for the structural part of the ESEM using obesity as a mediator. Only significant associations are included as arrows.
- The estimated regression coefficients for the direct, indirect and total effects of dietary patterns on obesity and the metabolic risk factors
- The estimated regression coefficients of the lifestyle and demographic confounders, modelling the direct effects of dietary patterns on obesity and on the metabolic risk factors.

5.1. ESEM using three dietary patterns and selected food variables by EFA

Table S4. Dietary factor loadings estimated by ESEM

	Women			Men		
	Diet 1	Diet 2	Diet 3	Diet 1	Diet 2	Diet 3
Cakes/Pastries	0.32	-0.03	0.05	0.30	0.05	0.05
Candy	0.27	-0.07	0.00	0.21	-0.05	0.01
Chips	0.29	0.00	0.02	0.27	-0.01	0.02
Rice/Pasta	0.27	0.13	0.02	0.21	0.26	-0.04
Compound meat dishes	0.44	0.04	0.10	0.50	0.10	0.08
Fish, unprocessed	-0.33	0.41	0.12	-0.30	0.36	0.23
Asian dishes	-0.02	0.29	-0.13	0.03	0.28	0.05
Chicken	0.12	0.50	0.02	0.10	0.34	0.02
Vegetables	-0.21	0.60	-0.07	-0.12	0.39	0.06
Bread	-0.05	-0.33	-0.02	-0.15	-0.28	-0.28
Porridge/Pancakes	0.07	-0.21	0.12	0.04	-0.11	0.11
Breakfast cereals, sweetened	0.02	-0.16	-0.02	-0.03	-0.02	-0.05
Meat spread	0.10	-0.04	0.06	0.08	-0.14	-0.15
Fish, processed	-0.03	0.08	0.32	-0.05	0.08	0.29
Meat, processed	0.15	-0.12	0.42	0.25	-0.22	0.45
Meat, red	0.03	0.16	0.48	0.09	0.08	0.44
Potato	-0.09	-0.12	0.37	-0.13	-0.07	0.38
Sauce etc.	0.15	0.13	0.32	0.16	0.15	0.33
Fish spread	-0.17	0.12	-0.02			
Breakfast cereals/Porridge, unsweetened	-0.09	0.13	-0.23			
Nuts	-0.11	0.15	-0.32			

Diet 1: Snacks and Meat pattern, Diet 2: Health-conscious pattern, Diet 3: Processed Dinner pattern

Table S5. Estimated regression coefficients of lifestyle and demographic variables in explaining three dietary patterns (selection of food variables)

	Women			Men		
	Diet 1	Diet 2	Diet 3	Diet 1	Diet 2	Diet 3
Age	−0.113*	−0.025*	0.029*	−0.106*	−0.027*	0.046*
Education level:						
Primary/partly secondary (ref)	0	0	0	0	0	0
Upper secondary	0.01	0.27*	−0.32*	0.20*	0.34*	−0.26*
Short tertiary	0.15	0.33*	−0.60*	0.18*	0.58*	−0.51*
Long tertiary	0.10	0.46*	−0.78*	0.25*	0.91*	−0.92*
Physical activity level:						
Sedentary (ref)	0	0	0	0	0	0
Light	−0.28*	0.24*	−0.38*	−0.24*	0.22*	−0.29*
High	−0.22*	0.49*	−0.67*	−0.30*	0.38*	−0.47*
Smoking status:						
Non-smokers (ref)	0	0	0	0	0	0
Smokers	−0.06	−0.20*	0.44*	−0.10	−0.29*	0.25*
Alcohol consumption	−0.13*	0.16*	0.04	−0.02	0.06*	0.04

Diet 1: Snacks and Meat pattern, Diet 2: Health-conscious pattern, Diet 3: Processed Dinner pattern

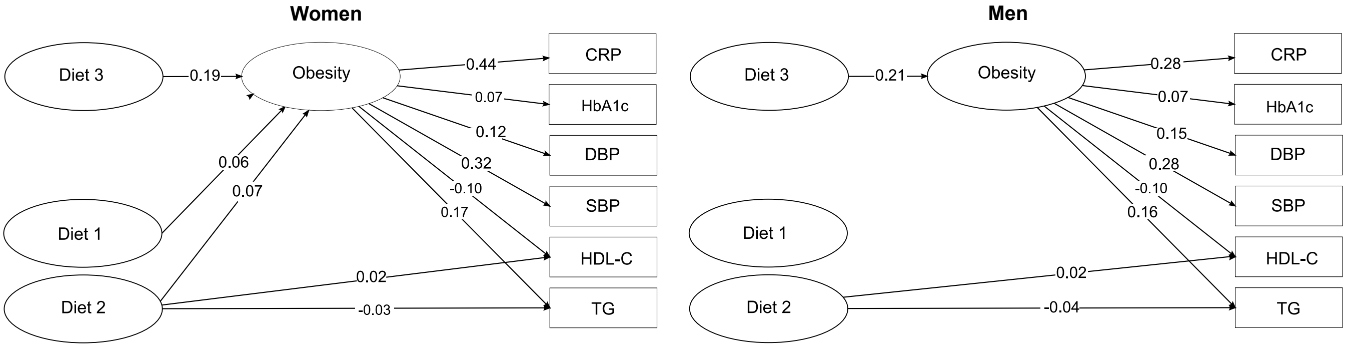

Figure S2: Estimated regression coefficients for the structural part of the ESEM using obesity as a mediator. The figures only include significant direct associations. Diet 1: Snacks and Meat pattern, Diet 2: Health-conscious pattern, Diet 3: Processed Dinner pattern (selection of food variables).

Table S6. The estimated regression coefficients for the direct, indirect and total effects of three dietary patterns on obesity and the metabolic risk factors (selection of food variables)

		Obesity	CRP	HDL-C	TG	HbA1c	SBP	DBP
Women:								
Diet 1	Direct	0.06*	0.03	−0.01	−0.01	−0.01	−0.01	−0.01
	Indirect		0.03*	−0.01*	0.01*	0.00*	0.02*	0.01*
	Total		0.05*	−0.02*	0.00	−0.01	0.01	0.00
Diet 2	Direct	0.07*	−0.03	0.02*	−0.03*	−0.02	−0.05	0.00
	Indirect		0.03*	−0.01*	0.01*	0.01*	0.02*	0.01*
	Total		0.00	0.01	−0.02	−0.01	−0.02	0.01
Diet 3	Direct	0.19*	0.03	0.00	0.02	0.00	0.03	0.00
	Indirect		0.08*	−0.02*	0.03*	0.01*	0.06*	0.02*
	Total		0.11*	−0.02*	0.05*	0.01	0.09*	0.02
Men:								
Diet 1	Direct	0.04	0.02	−0.01	0.01	−0.02	0.05	0.02
	Indirect		0.01	0.00	0.01	0.00	0.01	0.01
	Total		0.04	−0.01	0.02	−0.01	0.07	0.03
Diet 2	Direct	0.03	0.01	0.02*	−0.04*	−0.02	0.00	−0.01
	Indirect		0.01	0.00	0.00	0.00	0.01	0.00
	Total		0.01	0.01	−0.03*	−0.02	0.01	0.00
Diet 3	Direct	0.21*	0.00	0.01	−0.01	0.00	0.00	0.02
	Indirect		0.06*	−0.02*	0.03*	0.01*	0.06*	0.03*
	Total		0.06*	−0.01	0.02	0.01	0.06	0.05

CRP: C-reactive protein, HDL-C: HDL-cholesterol, TG: triglycerides, HbA1c: glycated hemoglobin, SBP: systolic blood pressure, DBP: diastolic blood pressure, Diet 1: Snacks and Meat pattern, Diet 2: Health-conscious pattern, Diet 3: Processed Dinner pattern
* P-value < 0.01

Table S7. Estimated regression coefficients of the lifestyle and demographic confounders, modelling the direct effects of three dietary patterns on obesity and the metabolic risk factors (selection of food variables)

	Obesity	CRP	HDL-C	TG	HbA1c	SBP	DBP
Women:							
Age	0.004	0.009*	0.005*	0.003	0.011*	0.079*	0.009*
Education level:							
Primary/partly secondary (ref)	0	0	0	0	0	0	0
Upper secondary	0.01	0.00	0.02	0.00	−0.03	−0.17	−0.03
Short tertiary	−0.13	−0.04	0.03	−0.04	−0.01	−0.26*	−0.05
Long tertiary	−0.22*	−0.04	0.03*	−0.03	−0.02	−0.30*	−0.07
Physical activity level:							
Sedentary (ref)	0	0	0	0	0	0	0
Light	−0.51*	−0.09	0.02	0.01	0.01	0.01	−0.03
High	−0.81*	−0.18*	0.06*	−0.05	0.02	−0.07	−0.07
Smoking status:							
Non-smokers (ref)	0	0	0	0	0	0	0
Smokers	−0.26*	0.18*	−0.08*	0.14*	0.12*	−0.16	0.00
Alcohol consumption	−0.06*	0.03	0.05*	−0.01	−0.04*	0.10*	0.12*
Men:							
Age	0.000	0.011*	0.005*	−0.008	0.007*	0.048*	0.000
Education level:							
Primary/partly secondary (ref)	0	0	0	0	0	0	0
Upper secondary	0.01	0.00	−0.02	0.05	−0.03	−0.10	−0.06
Short tertiary	0.04	−0.01	−0.04*	0.05	−0.04	−0.14	−0.07
Long tertiary	−0.19*	−0.05	−0.01	0.03	−0.05*	−0.22	−0.12
Physical activity level:							
Sedentary (ref)	0	0	0	0	0	0	0
Light	−0.47*	−0.12*	0.00	0.02	−0.03	0.17	0.05
High	−0.79*	−0.14*	0.05*	−0.05	−0.02	0.31*	0.06
Smoking status:							
Non-smokers (ref)	0	0	0	0	0	0	0
Smokers	−0.33*	0.28*	−0.07*	0.08*	0.12*	−0.22*	−0.09
Alcohol consumption	−0.05*	0.03*	0.03*	0.01	−0.01	0.11*	0.07*

CRP: C-reactive protein, HDL-C: HDL-cholesterol, TG: triglycerides, HbA1c: glycated hemoglobin, SBP: systolic blood pressure, DBP: diastolic blood pressure

* P-value < 0.01

5.2. ESEM using three dietary patterns and all aggregated food variables

Table S8. Dietary factor loadings estimated by ESEM

	Women			Men		
	Diet 1	Diet 2	Diet 3	Diet 1	Diet 2	Diet 3
Cakes/Pastries	0.29	-0.04	0.04	0.29	0.04	0.03
Candy	0.27	-0.07	0.02	0.22	-0.07	0.04
Chips	0.30	0.00	0.04	0.28	-0.02	0.05
Chocolate	0.16	-0.10	-0.06	0.13	-0.02	-0.04
Dessert	0.00	-0.10	0.09	0.04	-0.01	0.12
Rice/Pasta	0.27	0.10	0.00	0.24	0.22	-0.05
Asian dishes	-0.01	0.26	-0.15	0.06	0.29	0.01
Egg	-0.06	0.17	0.00	-0.02	0.03	0.00
Compound meat dishes	0.42	0.04	0.09	0.48	0.08	0.06
Porridge/Pancakes	0.05	-0.21	0.12	0.02	-0.10	0.11
Bread	-0.07	-0.29	0.01	-0.15	-0.31	-0.21
Butter and Margarine	0.03	-0.25	-0.01	-0.01	-0.32	-0.07
Cheese	-0.08	-0.07	-0.16	-0.09	-0.09	-0.20
Fish spread	-0.17	0.16	-0.01	-0.17	0.05	-0.02
Mayonnaise and Plant-based Oils	0.05	-0.04	0.10	0.07	-0.14	0.05
Meat spread	0.10	-0.01	0.07	0.08	-0.18	-0.10
Jam	-0.10	-0.25	-0.02	-0.15	-0.23	-0.10
Coffee	-0.06	0.00	0.16	-0.04	-0.05	0.15
Milk/Sugar for Coffee/Tea	-0.05	-0.04	-0.08	-0.06	0.00	-0.01
Milk	-0.05	-0.20	-0.07	-0.05	-0.22	-0.04
Juice	0.00	-0.08	-0.07	0.01	-0.03	-0.11
Soft drinks	0.17	0.01	0.06	0.17	-0.05	0.07
Fish, processed	-0.03	0.10	0.30	-0.07	0.13	0.27
Fish, unprocessed	-0.30	0.42	0.08	-0.27	0.40	0.18
Meat, processed	0.14	-0.08	0.43	0.20	-0.14	0.47
Meat, red	0.04	0.18	0.45	0.07	0.13	0.42
Chicken	0.14	0.48	-0.01	0.14	0.31	-0.01
Potato	-0.09	-0.08	0.36	-0.15	-0.01	0.39
Sauce etc.	0.16	0.14	0.31	0.17	0.17	0.33
Breakfast cereals/Porridge, unsweetened	-0.09	0.14	-0.23	-0.08	0.15	-0.18
Breakfast cereals, sweetened	0.01	-0.16	-0.02	-0.04	0.00	-0.06
Fruit	-0.10	0.24	0.04	-0.06	0.14	0.00
Nuts	-0.10	0.17	-0.29	-0.04	0.21	-0.15
Vegetables	-0.19	0.64	-0.10	-0.08	0.39	0.01
Yoghurt	-0.08	0.18	-0.21	-0.07	0.12	-0.07

Diet 1: Snacks and Meat pattern, Diet 2: Health-conscious pattern, Diet 3: Processed Dinner pattern

Table S9. Estimated regression coefficients of lifestyle and demographic variables in explaining three dietary patterns (all food variables)

	Women			Men		
	Diet 1	Diet 2	Diet 3	Diet 1	Diet 2	Diet 3
Age	−0.118*	−0.019*	0.030*	−0.109*	−0.009	0.040*
Education level:						
Primary/partly secondary (ref)	0	0	0	0	0	0
Upper secondary	0.06	0.22*	−0.38*	0.24*	0.28*	−0.30*
Short tertiary	0.21*	0.25*	−0.71*	0.23*	0.52*	−0.63*
Long tertiary	0.15	0.33*	−1.03*	0.34*	0.79*	−1.10*
Physical activity level:						
Sedentary (ref)	0	0	0	0	0	0
Light	−0.35*	0.23*	−0.39*	−0.24*	0.23*	−0.34*
High	−0.27*	0.46*	−0.70*	−0.27*	0.39*	−0.58*
Smoking status:						
Non-smokers (ref)	0	0	0	0	0	0
Smokers	−0.06	−0.18*	0.51*	−0.09	−0.33*	0.37*
Alcohol consumption	−0.08*	0.22*	0.05	0.00	0.09*	0.06*

Diet 1: Snacks and Meat pattern, Diet 2: Health-conscious pattern, Diet 3: Processed Dinner pattern
* P-value < 0.01

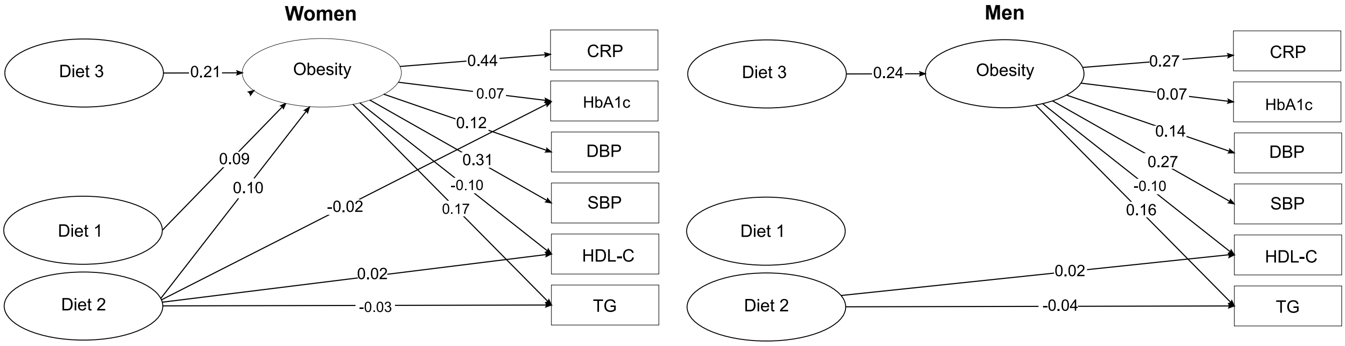

Figure S3: Estimated regression coefficients for the structural part of the ESEM using obesity as a mediator. The figures only include significant direct associations. Diet 1: Snacks and Meat pattern, Diet 2: Health-conscious pattern, Diet 3: Processed Dinner pattern (all food variables).

Table S10. The estimated regression coefficients for the direct, indirect and total effects of three dietary patterns on obesity and the metabolic risk factors (all food variables)

		Obesity	CRP	HDL-C	TG	HbA1c	SBP	DBP
Women:								
Diet 1	Direct	0.09*	0.04	−0.01	−0.01	−0.01	−0.02	−0.02
	Indirect		0.04*	−0.01*	0.02*	0.01*	0.03*	0.01*
	Total		0.08*	−0.02*	0.01	−0.01	0.01	−0.01
Diet 2	Direct	0.10*	−0.02	0.02*	−0.03*	−0.02*	−0.05	0.00
	Indirect		0.04*	−0.01*	0.02*	0.01*	0.03*	0.01*
	Total		0.02	0.01	−0.01	−0.01	−0.02	0.01
Diet 3	Direct	0.21*	0.02	0.00	0.02	0.00	0.03	0.00
	Indirect		0.09*	−0.02*	0.04*	0.02*	0.07*	0.03*
	Total		0.11*	−0.02*	0.05*	0.01	0.10*	0.03
Men:								
Diet 1	Direct	0.05	0.04	−0.01	0.02	−0.02	0.05	0.02
	Indirect		0.01	−0.01	0.01	0.00	0.01	0.01
	Total		0.05*	−0.01	0.02	−0.01	0.06	0.02
Diet 2	Direct	0.05	−0.01	0.02*	−0.04*	−0.02	0.01	0.00
	Indirect		0.01	−0.01	0.01	0.00	0.01	0.01
	Total		0.00	0.01	−0.04*	−0.01	0.02	0.01
Diet 3	Direct	0.24*	0.01	0.00	0.00	0.00	0.02	0.02
	Indirect		0.07*	−0.02*	0.04*	0.02*	0.07*	0.03*
	Total		0.08*	−0.02*	0.04*	0.01	0.08	0.06*

CRP: C-reactive protein, HDL-C: HDL-cholesterol, TG: triglycerides, HbA1c: glycated hemoglobin, SBP: systolic blood pressure, DBP: diastolic blood pressure, Diet 1: Snacks and Meat pattern, Diet 2: Health-conscious pattern, Diet 3: Processed Dinner pattern
* P-value < 0.01

Table S11. Estimated regression coefficients of the lifestyle and demographic confounders, modelling the direct effects of three dietary patterns on obesity and the metabolic risk factors (all food variables)

	Obesity	CRP	HDL-C	TG	HbA1c	SBP	DBP
Women:							
Age	0.007	0.011*	0.005*	0.004*	0.011*	0.078*	0.008*
Education level:							
Primary/partly secondary (ref)	0	0	0	0	0	0	0
Upper secondary	0.02	0.00	0.02	0.00	−0.03	−0.16	−0.02
Short tertiary	−0.11	−0.04	0.03	−0.04	−0.01	−0.25*	−0.05
Long tertiary	−0.18*	−0.05	0.04*	−0.03	−0.02	−0.29*	−0.06
Physical activity level:							
Sedentary (ref)	0	0	0	0	0	0	0
Light	−0.50*	−0.09	0.02	0.01	0.01	0.01	−0.03
High	−0.80*	−0.18*	0.06*	−0.05	0.02	−0.07	−0.07
Smoking status:							
Non-smokers (ref)	0	0	0	0	0	0	0
Smokers	−0.28*	0.18*	−0.08*	0.14*	0.12*	−0.17	−0.01
Alcohol consumption	−0.08*	0.03	0.05*	−0.01	−0.04*	0.10*	0.12*
Men:							
Age	0.000	0.011*	0.004*	−0.007*	0.008*	0.047*	0.000
Education level:							
Primary/partly secondary (ref)	0	0	0	0	0	0	0
Upper secondary	0.02	0.01	−0.02	0.05	−0.03	−0.10	−0.06
Short tertiary	0.07	0.01	−0.04*	0.06	−0.05	−0.14	−0.07
Long tertiary	−0.15	−0.03	−0.01	0.04	−0.05*	−0.22	−0.12
Physical activity level:							
Sedentary (ref)	0	0	0	0	0	0	0
Light	−0.45*	−0.11*	0.00	0.02	−0.03	0.16	0.05
High	−0.76*	−0.12*	0.05*	−0.04	−0.02	0.30*	0.06
Smoking status:							
Non-smokers (ref)	0	0	0	0	0	0	0
Smokers	−0.36*	0.27*	−0.07*	0.07*	0.12*	−0.22*	−0.09
Alcohol consumption	−0.04*	0.04*	0.03*	0.01	−0.01	0.11*	0.07*

CRP: C-reactive protein, HDL-C: HDL-cholesterol, TG: triglycerides, HbA1c: glycated hemoglobin, SBP: systolic blood pressure, DBP: diastolic blood pressure

5.3. ESEM using four dietary patterns and all aggregated food variables

Unlike the model using selected food variables, using four dietary patterns and all food variables resulted in four similar patterns instead of three. Consequently, both patterns were labeled Diet 4a: the porridge pattern.

Table S12. Dietary factor loadings estimated by ESEM

	Women				Men			
	Diet 1	Diet 2	Diet 3	Diet 4a	Diet 1	Diet 2	Diet 3	Diet 4a
Cakes/Pastries	0.31	0.07	0.09	0.24	0.27	0.07	0.00	0.33
Candy	0.27	-0.06	0.00	0.06	0.21	-0.05	0.02	0.12
Chips	0.29	-0.08	0.02	-0.08	0.27	-0.01	0.03	0.04
Chocolate	0.15	-0.06	-0.09	0.06	0.12	-0.01	-0.06	0.18
Dessert	0.00	-0.03	0.09	0.19	0.05	-0.01	0.11	0.26
Rice/Pasta	0.27	0.11	0.04	0.01	0.21	0.24	-0.06	0.04
Asian dishes	0.02	0.34	-0.04	-0.03	0.04	0.29	0.02	0.02
Egg	-0.06	0.03	0.01	-0.27	-0.02	0.03	0.01	-0.21
Compound meat dishes	0.43	0.05	0.13	0.10	0.46	0.11	0.02	0.20
Porridge/Pancakes	0.06	-0.04	0.12	0.41	0.03	-0.11	0.09	0.43
Bread	-0.10	-0.35	-0.12	-0.01	-0.15	-0.32	-0.22	-0.19
Butter and Margarine	0.01	-0.28	-0.12	0.01	0.01	-0.31	-0.09	-0.09
Cheese	-0.08	-0.07	-0.20	-0.06	-0.09	-0.08	-0.20	-0.08
Fish spread	-0.18	0.01	-0.01	-0.29	-0.16	0.04	0.00	-0.31
Mayonnaise and Plant-based Oils	0.03	-0.17	0.05	-0.13	0.08	-0.15	0.04	-0.19
Meat spread	0.07	-0.21	0.00	-0.26	0.08	-0.19	-0.12	-0.28
Jam	-0.10	-0.14	-0.08	0.21	-0.15	-0.24	-0.12	0.16
Coffee	-0.07	-0.07	0.14	-0.04	-0.02	-0.06	0.15	-0.09
Milk/Sugar for Coffee/Tea	-0.05	-0.01	-0.09	0.00	-0.06	0.00	0.00	0.01
Milk	-0.05	-0.09	-0.11	0.16	-0.03	-0.21	-0.05	0.04
Juice	0.00	-0.05	-0.10	0.03	0.00	-0.02	-0.12	0.01
Soft drinks	0.16	-0.05	0.05	-0.05	0.16	-0.04	0.05	0.00
Fish, processed	-0.03	0.04	0.34	0.05	-0.05	0.10	0.28	0.08
Fish, unprocessed	-0.28	0.34	0.21	-0.19	-0.26	0.36	0.23	-0.06
Meat, processed	0.12	-0.20	0.39	0.08	0.22	-0.16	0.44	0.04
Meat, red	0.03	0.02	0.49	-0.04	0.09	0.11	0.43	-0.02
Chicken	0.15	0.36	0.12	-0.28	0.12	0.32	0.00	-0.05
Potato	-0.10	-0.15	0.33	0.10	-0.12	-0.05	0.40	0.05
Sauce etc.	0.15	0.01	0.34	-0.04	0.18	0.15	0.33	0.03
Breakfast cereals/Porridge, unsweetened	-0.06	0.30	-0.14	0.08	-0.10	0.16	-0.17	0.11
Breakfast cereals, sweetened	0.02	0.02	-0.01	0.33	-0.04	0.00	-0.07	0.35
Fruit	-0.10	0.17	0.10	-0.15	-0.06	0.14	0.02	-0.08
Nuts	-0.07	0.28	-0.21	-0.04	-0.06	0.22	-0.13	0.03
Vegetables	-0.16	0.55	0.09	-0.34	-0.10	0.38	0.04	-0.07
Yoghurt	-0.06	0.25	-0.13	-0.05	-0.08	0.12	-0.06	0.02

Diet 1: Snacks and Meat pattern, Diet 2: Health-conscious pattern, Diet 3: Processed Dinner pattern, Diet 4a: Porridge pattern

Table S13. Estimated regression coefficients of lifestyle and demographic variables in explaining four dietary patterns (all food variables)

	Women				Men			
	Diet 1	Diet 2	Diet 3	Diet 4a	Diet 1	Diet 2	Diet 3	Diet 4a
Age	−0.122*	−0.009	0.022*	0.026*	−0.116*	−0.018	0.046*	0.018
Education level:								
Primary/partly secondary (ref)	0	0	0	0	0	0	0	
Upper secondary	0.09	0.28*	−0.30*	−0.17*	0.24*	0.31*	−0.29*	−0.10
Short tertiary	0.24*	0.43*	−0.59*	−0.15	0.20	0.57*	−0.60*	−0.15
Long tertiary	0.17	0.69*	−0.84*	−0.05	0.13	0.86*	−1.07*	0.19
Physical activity level:								
Sedentary (ref)	0	0	0	0	0	0	0	
Light	−0.30*	0.36*	−0.29*	−0.08	−0.33*	0.23*	−0.31*	0.08
High	−0.25*	0.72*	−0.50*	−0.08	−0.42*	0.41*	−0.54*	0.14
Smoking status:								
Non-smokers (ref)	0	0	0	0	0	0	0	
Smokers	−0.03	−0.51*	0.38*	−0.23*	0.05	−0.35*	0.36*	−0.24
Alcohol consumption	−0.04	0.03	0.08*	−0.34*	0.08*	0.10*	0.07*	−0.21*

Diet 1: Snacks and Meat pattern, Diet 2: Health-conscious pattern, Diet 3: Processed Dinner pattern, Diet 4a: Porridge pattern
* P-value < 0.01.

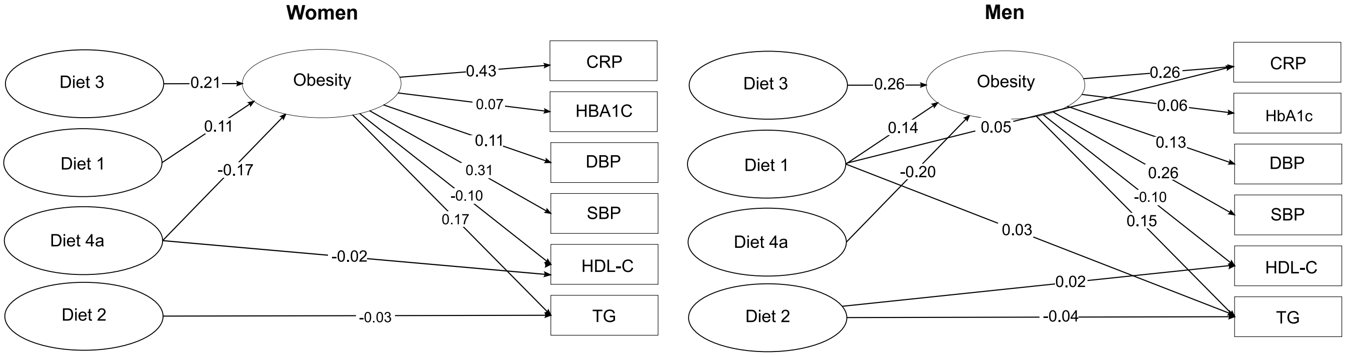

Figure S4: Estimated regression coefficients for the structural part of the ESEM using obesity as a mediator. The figures only include significant direct associations. Diet 1: Snacks and Meat pattern, Diet 2: Health-conscious pattern, Diet 3: Processed Dinner pattern, Diet 4a: Porridge pattern (all food variables).

Table S14. The estimated regression coefficients for the direct, indirect and total effects of four dietary patterns on obesity and the metabolic risk factors (all food variables)

		Obesity	CRP	HDL-C	TG	HbA1c	SBP	DBP
Women:								
Diet 1	Direct	0.11*	0.03	−0.01	−0.01	−0.02	−0.01	−0.01
	Indirect		0.05*	−0.01*	0.02*	0.01*	0.03*	0.01*
	Total		0.08*	−0.02*	0.01	−0.01	0.02	0.00
Diet 2	Direct	−0.07	−0.03	0.01	−0.03*	−0.01	−0.06	−0.01
	Indirect		−0.03	0.01	−0.01	−0.01	−0.02	−0.01
	Total		−0.06*	0.01*	−0.05*	−0.02*	−0.08	−0.02
Diet 3	Direct	0.21*	0.01	0.01	0.01	−0.01	0.02	0.00
	Indirect		0.09*	−0.02*	0.04*	0.02*	0.06*	0.02*
	Total		0.11*	−0.01*	0.04*	0.01	0.08	0.03
Diet 4a	Direct	−0.17*	0.01	−0.02*	0.01	0.01	0.01	−0.02
	Indirect		−0.07*	0.02*	−0.03*	−0.01*	−0.05*	−0.02*
	Total		−0.06*	0.00	−0.02	0.00	−0.05	−0.04
Men:								
Diet 1	Direct	0.14*	0.05*	−0.01	0.03*	−0.01	0.06	0.03
	Indirect		0.04*	−0.01*	0.02*	0.01*	0.04*	0.02*
	Total		0.08*	−0.02*	0.05*	0.00	0.10*	0.05
Diet 2	Direct	0.04	−0.01	0.02*	−0.04*	−0.02	0.01	0.00
	Indirect		0.01	0.00	0.01	0.00	0.01	0.01
	Total		0.00	0.01	−0.04*	−0.01	0.02	0.00
Diet 3	Direct	0.26*	0.01	0.01	0.00	0.00	0.02	0.03
	Indirect		0.07*	−0.02*	0.04*	0.02*	0.07*	0.03*
	Total		0.08*	−0.02*	0.04*	0.01	0.08	0.06*
Diet 4a	Direct	−0.20*	−0.02	−0.01	−0.02	−0.01	−0.03	−0.03
	Indirect		−0.05*	0.02*	−0.03*	−0.01*	−0.05*	−0.03*
	Total		−0.07*	0.01	−0.05*	−0.03*	−0.08	−0.05*

CRP: C-reactive protein, HDL-C: HDL-cholesterol, TG: triglycerides, HbA1c: glycated hemoglobin, SBP: systolic blood pressure, DBP: diastolic blood pressure, Diet 1: Snacks and Meat pattern, Diet 2: Health-conscious pattern, Diet 3: Processed Dinner pattern, Diet 4a: Porridge pattern

* P-value < 0.01.

1

Table S15. Estimated regression coefficients of the lifestyle and demographic confounders, modelling the direct effects of four dietary patterns on obesity and the metabolic risk factors (all food variables)

	Obesity	CRP	HDL-C	TG	HbA1c	SBP	DBP
Women:							
Age	0.013*	0.011*	0.005*	0.004*	0.010*	0.079*	0.009*
Education level:							
Primary/partly secondary (ref)	0	0	0	0	0	0	0
Upper secondary	0.01	0.00	0.02	0.00	−0.03	−0.16	−0.02
Short tertiary	−0.11	−0.04	0.03	−0.04	−0.01	−0.25*	−0.05
Long tertiary	−0.15*	−0.05	0.04*	−0.03	−0.02	−0.29*	−0.06
Physical activity level:							
Sedentary (ref)	0	0	0	0	0	0	0
Light	−0.49*	−0.09	0.02	0.01	0.01	0.01	−0.03
High	−0.76*	−0.18*	0.06*	−0.05	0.02	−0.06	−0.07
Smoking status:							
Non-smokers (ref)	0	0	0	0	0	0	0
Smokers	−0.35*	0.18*	−0.08*	0.14*	0.13*	−0.17	−0.02
Alcohol consumption	−0.12*	0.03	0.05*	−0.01	−0.03*	0.09	0.11*
Men:							
Age	0.013*	0.013*	0.005*	−0.006	0.008*	0.050*	0.002
Education level:							
Primary/partly secondary (ref)	0	0	0	0	0	0	0
Upper secondary	−0.02	0.00	−0.02	0.05	−0.03	−0.10	−0.07
Short tertiary	0.02	0.00	−0.04*	0.05	−0.05	−0.15	−0.08
Long tertiary	−0.11	−0.02	−0.01	0.05	−0.05*	−0.21	−0.11
Physical activity level:							
Sedentary (ref)	0	0	0	0	0	0	0
Light	−0.42*	−0.11	0.00	0.03	−0.03	0.17	0.06
High	−0.71*	−0.12*	0.05*	−0.04	−0.02	0.31*	0.06
Smoking status:							
Non-smokers (ref)	0	0	0	0	0	0	0
Smokers	−0.44*	0.26*	−0.07*	0.06*	0.12*	−0.24*	−0.11
Alcohol consumption	−0.02	0.03	0.03*	0.00	−0.01	0.10*	0.06*

CRP: C-reactive protein, HDL-C: HDL-cholesterol, TG: triglycerides, HbA1c: glycated hemoglobin, SBP: systolic blood pressure, DBP: diastolic blood pressure
* P-value < 0.01.

6 GOODNESS OF FIT MEASURES FOR ALL FITTED ESEMS

In summary, four ESEMs have been investigated in detail for both women and men. These include models using three or four dietary patterns, either using the selected subset of aggregated food variables found by EFA or all of the 35 aggregated food variables. The main model using 4 dietary patterns and selected food variables based on EFA was the only model giving a CFI value above 0.90.

Table S16. Goodness of fit measures for the four fitted models for both women and men.

		CFI		RMSEA		SRMR	
		Women	Men	Women	Men	Women	Men
3 dietary patterns:	All food variables	0.765	0.734	0.045	0.048	0.035	0.038
	Selection of variables	0.876	0.877	0.044	0.047	0.032	0.033
4 dietary patterns:	All food variables	0.801	0.784	0.042	0.044	0.030	0.030
	Selection of variables	0.908	0.917	0.039	0.041	0.025	0.026

CFI: comparative fit index, RMSEA: root mean square error of approximation, SRMR: standardized root mean square residual
